# Colloidal solutions of luminescent porous silicon clusters with different cluster sizes

**DOI:** 10.1186/1556-276X-9-478

**Published:** 2014-09-09

**Authors:** Kateřina Herynková, Egor Podkorytov, Miroslav Šlechta, Ondřej Cibulka, Jindřich Leitner, Ivan Pelant

**Affiliations:** 1 Department of Thin Films and Nanostructures, Institute of Physics, Academy of Sciences of the Czech Republic, v.v.i., Cukrovarnická 10, 162 53 Prague, Czech Republic; 2 Department of Solid State Engineering, Institute of Chemical Technology, Prague 166 28, Czech Republic

**Keywords:** Nanocrystalline silicon, Porous silicon, Cluster size, Luminescent markers

## Abstract

Silicon nanocrystals (Si-ncs) are promising for biological studies due to their supposed low cytotoxicity, good biocompatibility and biodegradability in living organisms. However, the bioresearchers' focus on Si-ncs has lasted only for a few recent years, and detailed studies of the interaction of various types of Si-ncs with biological environment are still rare. Suitable size and solubility of the Si-ncs in water-based isotonic solutions are important towards bringing the nanocrystals inside the living cells. We have prepared colloidal solutions of luminescent porous silicon of different cluster sizes in methanol, water and phosphate-buffered saline (PBS). By combination of ultrasonic treatment with filtration, we have obtained two different silicon cluster sizes in methanol (120 and 525 nm) and three different cluster sizes (85, 210 and 1,500 nm) in PBS. Nanoclusters of heavily oxidized porous silicon are hydrophilic and well soluble in water and/or PBS. They can be further used for studies on the biocompatibility of these materials and may be potentially employed as luminescent markers in living cells in biological research.

**PACS:**

78.67.Rb; 78.67.-n; 87.85.Qr; 87.85.Rs; 81.07.-b

## Background

Luminescent porous silicon, together with other nanocrystalline silicon forms, has been widely studied for the last twenty years due to its potential use in silicon nanophotonics or solar energy conversion [1]. In biological studies, however, nanocrystalline silicon waited for its chance till the late 2000’s when, until that time, mostly used CdSe quantum dots were proven to be considerably cytotoxic [2,3], and the attention has been drawn to alternative nanocrystalline materials such as Si-ncs [4] or nanodiamonds. [5] Since then, Si-ncs have been considered promising for many biological applications - as fluorescent labels, biological sensors, drug delivery systems or scaffold for various tissues [6,7]. The main advantages of Si-ncs are low cytotoxicity [8], easy functionalization [9], efficient photoluminescence [10] and biodegradability [11-13]. Photo- and sonosensitizing properties of porous silicon were successfully employed in simultaneous cancer therapy and diagnostics [14-16].

A hot topic in the biological research is the development of techniques for *in vivo* investigation of biological processes on subcellular or a single-molecule level. For example, porous silicon quantum dots were recently used in confocal fluorescence microscopy at the single-cell level for bone resorption studies [17].

However, the toxicity of porous silicon for the living cells neither has been sufficiently determined yet, nor its potential to be used as luminescent markers on subcellular level has been evaluated. The aim of this work is to prepare colloidal solutions of luminescent silicon nanoclusters with several different and known cluster sizes of the order of tens to hundreds of nanometers which can be further used for the biological studies of cytotoxicity. Therefore, phosphate-buffered saline (PBS) was used as a preferred solvent because it is a non-toxic and isotonic buffer solution commonly used in biological research. By combination of ultrasonic treatment, filtration with the syringe filters and ageing of the samples, we obtained three suitable colloidal solutions of luminescent silicon clusters of the sizes of 85, 210 and 1,500 nm.

## Methods

**Figure 1:**
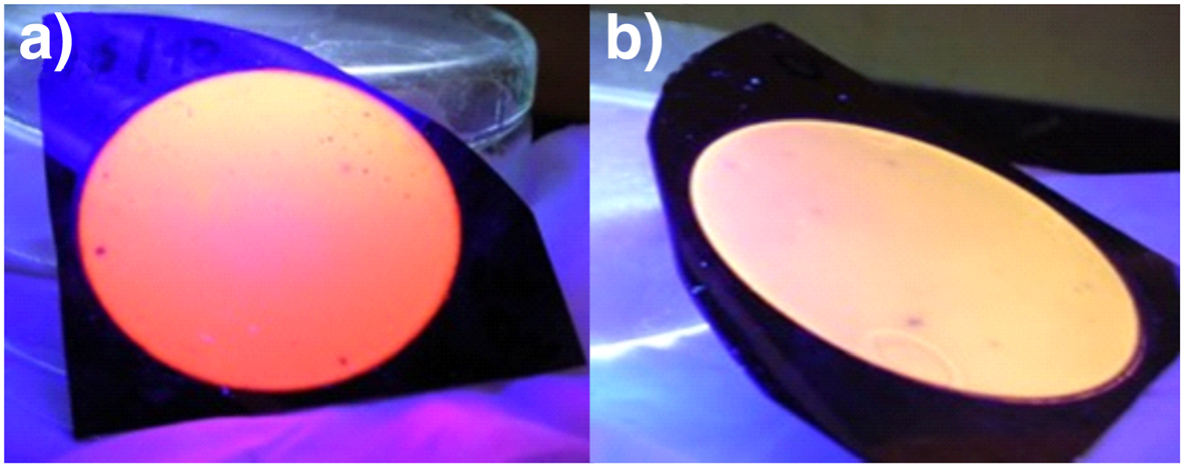
Freshly etched samples of (a) ‘standard’ and (b) ‘white’ porous silicon under illumination with a UV lamp.

Two types of porous silicon powders were used for this study. The first ‘standard’ porous silicon was prepared by standard anodic electrochemical etching of a p-type [100] silicon wafer (boron doping, resistivity 0.06 to 0.1 Ω cm) in a 1:3 aqueous-HF (49%) solution in ethanol for 2 h at current density 1.6 mA/cm^2^. During etching of the second type of porous silicon (denoted as ‘white’ due to its colour), higher current density (2.5 mA/cm^2^) was used, a small amount of 30% hydrogen peroxide was added to the electrolyte and the resulting porous silicon sample was then post-etched in a hydrogen peroxide bath for 16 min. Figure 1 shows photographs of both types of freshly etched samples luminescing under illumination with UV lamp. Porous silicon powders were then mechanically scraped from the etched wafers and dissolved in methanol, water or PBS (starting concentration was always 2 mg of Si-ncs powders in 1 cm^3^ of solvent) and ultrasonicated for 1 h in order to break big cluster agglomerates.

To access the size of Si nanoparticles and/or their clusters, dynamic light scattering (DLS) in colloidal suspensions was applied using a Zetasizer Nano S (Malvern, Grovewood Road, Malvern, UK), the samples being illuminated by the 633-nm line of a HeNe laser and signal was detected in a backscattering geometry. The dynamic nanocluster sizes were calculated by Zetasizer software at temperature 25°C using refractive indices 3.5 for Si, 1.326 for methanol and 1.330 for water/PBS, solvent viscosities 0.05476 cP for methanol and 0.8872 cP for water/PBS and data were processed in general purpose (normal) resolution mode.

Fourier transform infrared (FTIR) spectra were measured by a Thermo Scientific Nicolet iN10 infrared microscope (Thermo Scientific, Waltham, MA, USA). X-ray diffraction (XRD) was recorded by the Empyrean diffractometer system in capillary spinner configuration with copper XRD tube. Photoluminescence was excited by a 325-nm line of a continuous wave HeCd laser and detected by imaging spectrograph connected to an Andor CCD camera (Andor, Springvale Business Park, Belfast, UK). All spectra were recorded at room temperature and corrected for the spectral response of the detection path.

## Results and discussion

**Figure 2:**
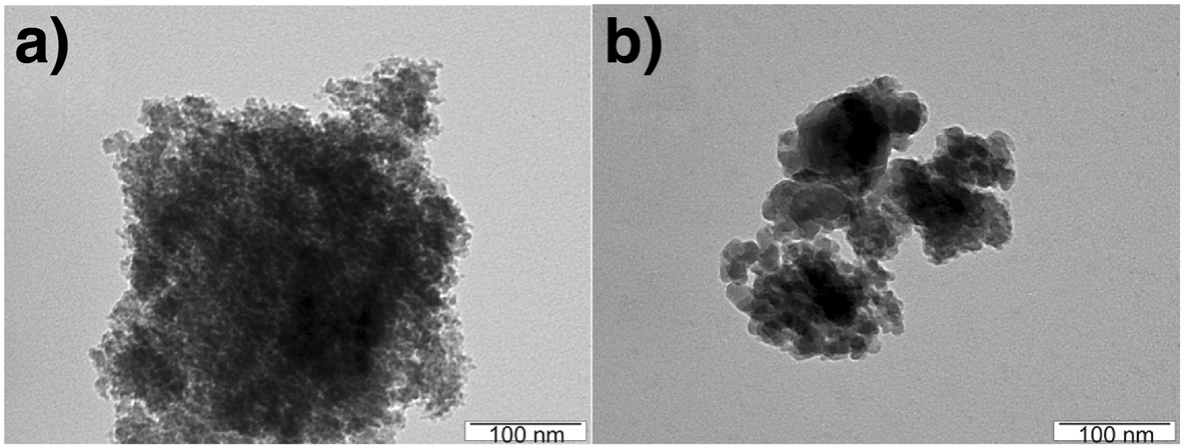
**TEM images of the porous silicon nanoclusters used for dissolving in colloidal dispersions. (a)** ‘Standard’ porous silicon and **(b)** ‘white’ porous silicon.

**Figure 3:**
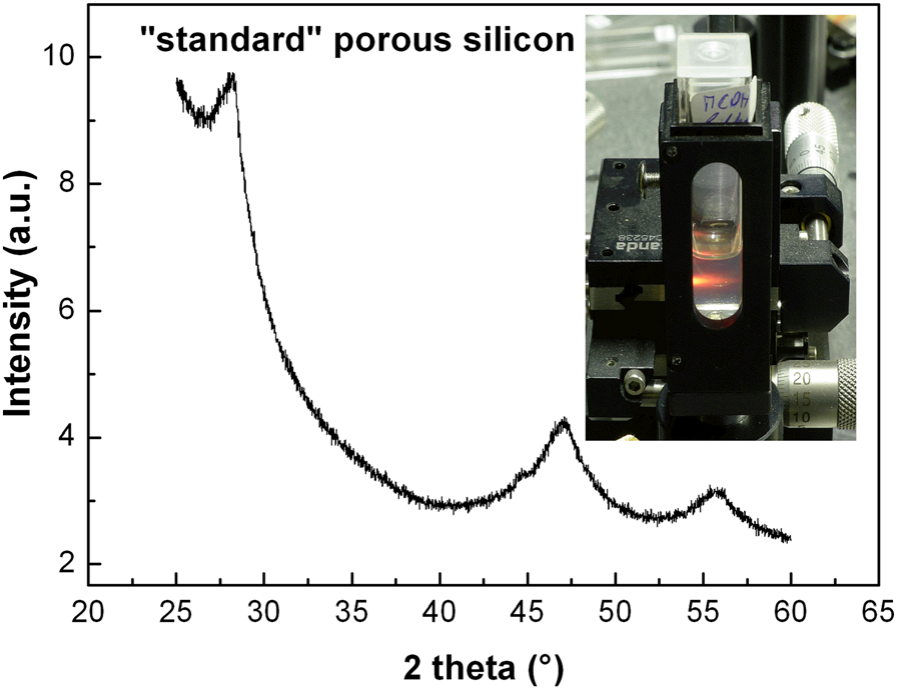
**XRD of ‘standard’ porous silicon manifesting presence of the crystalline silicon phase.** Estimated nanocrystal size from the Scherrer equation is 4 nm. Inset shows a photograph of final colloidal solution - stable ‘standard’ porous silicon nanoparticles in methanol. Sample is filtered by the 1-μm syringe filter and shows strong orange photoluminescence under illumination by 325-nm line of the HeCd laser.

Figure 2 shows the transmission electron microscopy (TEM) images of both types of porous silicon powders. Both exhibit a similar cauliflower structure of several-hundred-nanometer sized clusters. These clusters are composed of smaller (2 to 4 nm) luminescent silicon nanocrystals. The presence of silicon nanocrystals can be revealed by the XRD - an example of the XRD data of the ‘standard’ porous silicon powder is shown in Figure 3. Broad reflections of the crystalline silicon phase are apparent in the recording. Peak broadening is due to several effects: defects, stress and particle size. Scherrer equation gives silicon nanoparticle size of 4 nm. Detailed high-resolution transmission electron microscopy of the prepared silicon nanocrystals can be found in [18] and more data on the samples' structure and photoluminescence properties can be found in [19-22], listing our previous studies of photoluminescence in colloidal solutions with porous silicon.

**Figure 4:**
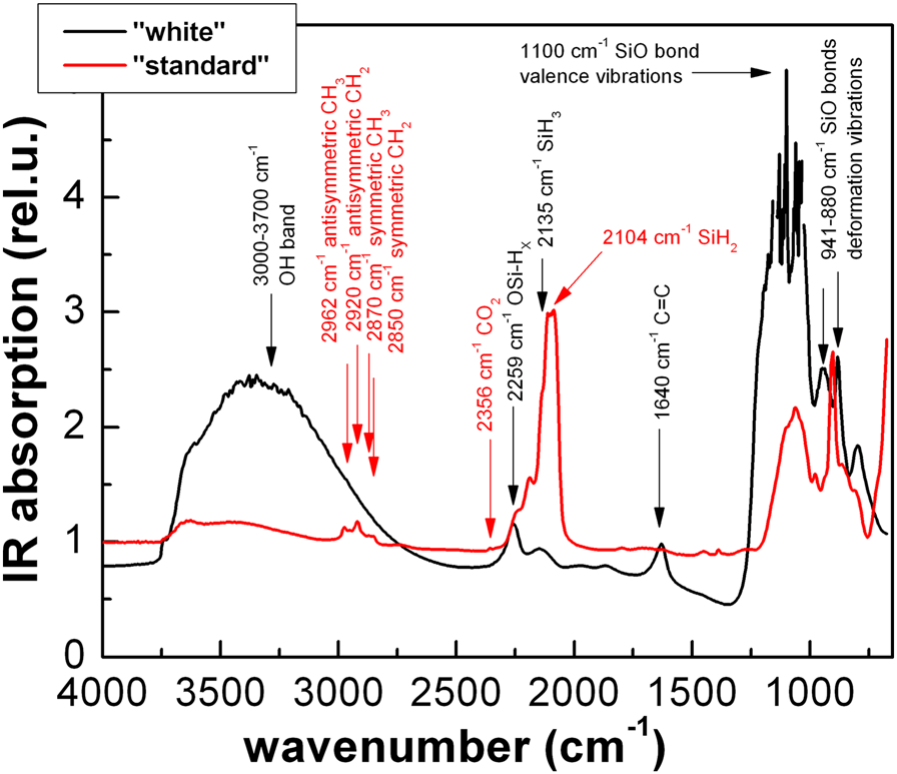
Comparison of FTIR spectra of ‘standard’ and ‘white’ porous silicon.

**Figure 5:**
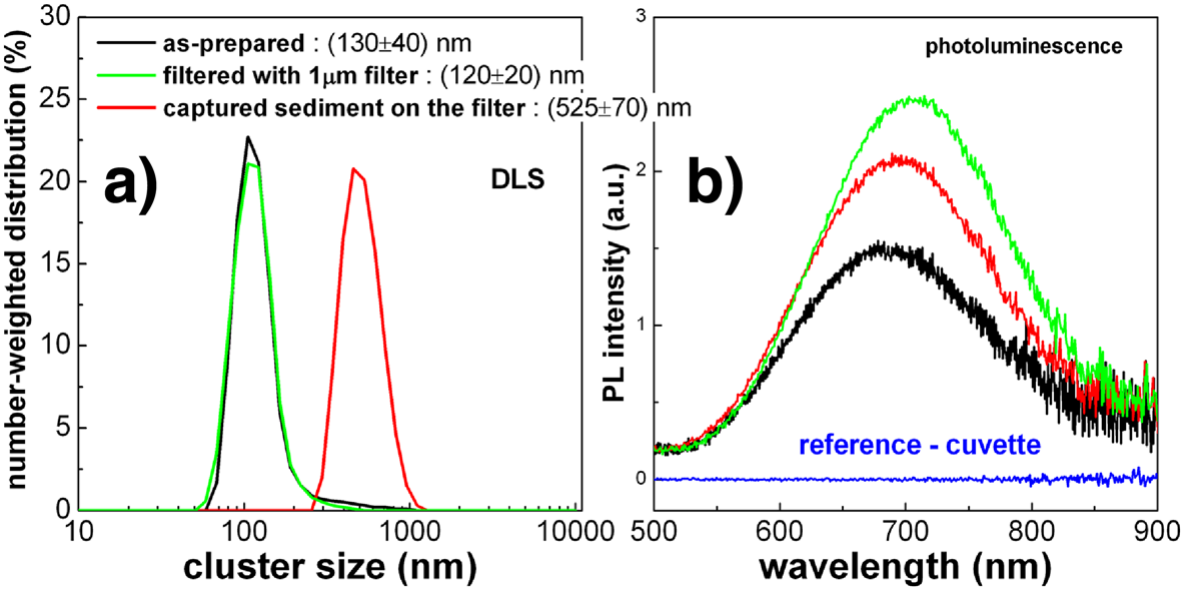
**Colloidal solution of standard porous silicon in methanol. (a)** Size distribution of the as**-**prepared sample (black line), sample filtered by the 1-μm syringe filter (green line) and sample made from the sediment remained on the filter (red line) obtained by dynamic light scattering (DLS). **(b)** Photoluminescence spectra of all three samples.

The difference between ‘standard’ and ‘white’ porous silicon powders emerges from a comparison of the FTIR spectra plotted in Figure 4. White porous silicon, with respect to a standard sample, exhibits an additional broad band between 3,000 and 3,500 cm^−1^ attributed to -OH groups, together with a series of peaks at around 1,000 to 1,100 cm^−1^ due to vibrations of Si-O bonds. Therefore, silicon nanocrystals composing white porous silicon have stronger oxidized surface than ‘standard’ ones and their nanocrystal core is smaller which implies a blue shift of photoluminescence (PL) - the PL of standard porous silicon is red (peaked at 700 nm), while the PL of white porous silicon is light orange and centred at around 600 nm. The last but crucial difference is that unlike hydrophobic standard porous silicon (not soluble in water-based solutions), the oxidized surface makes white porous silicon hydrophilic and easily soluble in water and water-based isotonic solvents.The as-prepared colloidal solutions of porous silicon powders contain generally nanoclusters of different sizes. Typically, 60% to 80% nanoclusters have a size between 100 and 200 nm but also 500 to 600 nm, and several micron-sized agglomerates are present in the solutions which can be separated by filtration. Results of filtration of standard porous silicon in methanol dispersion are summarized in Figure 5. By a straightforward (however, multiple in order to well separate the nanoclusters by their size) filtration of the solution with 1-μm syringe filters and gathering the sediment on the filter, we could easily separate nanoclusters of two sizes: 120 and 525 nm (estimated concentrations are 50 or 1.6 mg/ml, respectively). Both cluster sizes have comparable luminescence intensity which is situated in the red spectral region at around 700 nm. Similar results were obtained in the case of filtration of methanol solution of white porous silicon powder.

**Figure 6:**
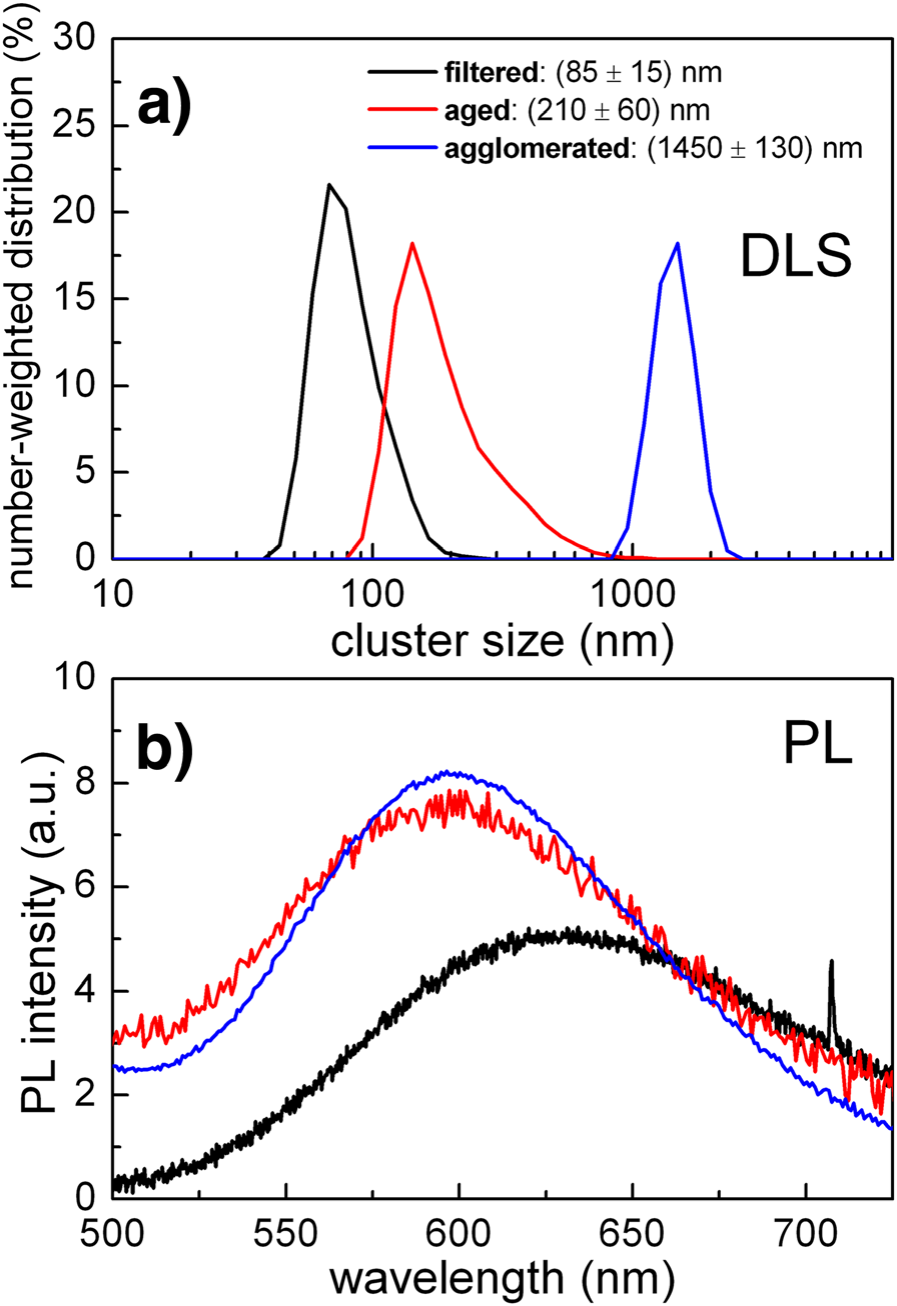
**Results of DLS and photoluminescence measurements in colloidal solution of** ‘**white**’ **porous silicon in PBS. (a)** Nanocluster size distribution of three different types of samples: black line - solution filtered with 1-μm syringe filter, red line - sample aged at ambient conditions and blue line - agglomerated sample (see text for details). **(b)** Corresponding photoluminescence spectra.

**Figure 7:**
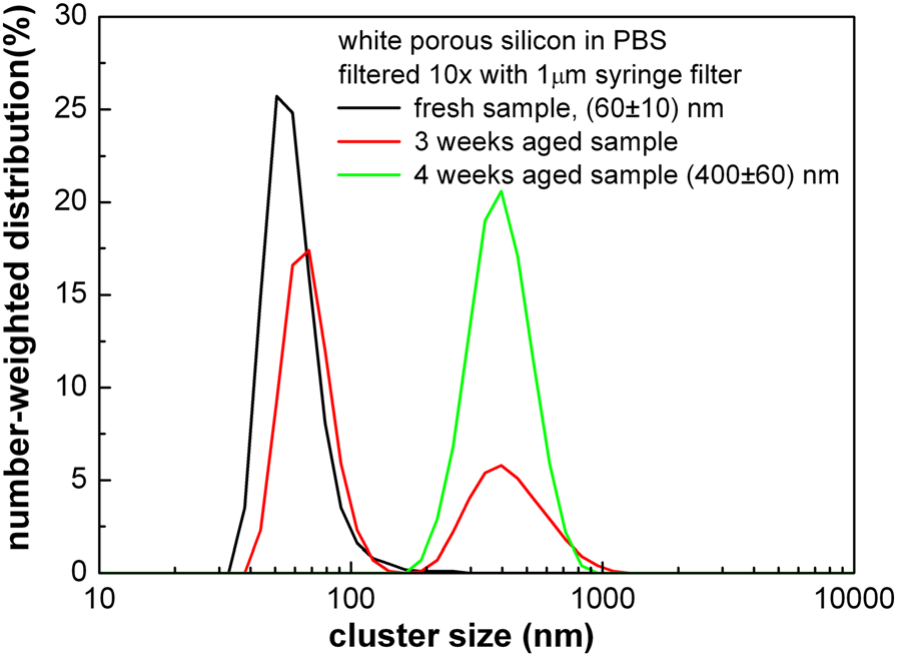
**Ageing of the** ‘**white**’ **porous silicon in PBS solution.** DLS measurement of the freshly filtered sample and sample aged for 3 and 4 weeks at ambient conditions.

As already mentioned, standard porous silicon is well known to be a hydrophobic material, soluble neither in water nor in PBS. In order to prepare silicon nanoclusters in water or PBS solution, we used white porous silicon powder which is hydrophilic. In this case, however, the filtration did not appear to be an efficient manner to separate nanocrystals of different sizes; despite multiple filtrations, a big fraction of the smallest nanoclusters still did not pass through the filter and only approximately 5% of nanoclusters smaller than 100 nm were gained. Another approach had to be chosen to obtain different sizes of nanoclusters; the results discussed below are common for both water and PBS solutions.Figure 6 shows the results of DLS and photoluminescence experiments performed on three types of samples: (i) solution filtered with a syringe filter, (ii) sample ultrasonicated and aged for 7 days at ambient conditions and (iii) agglomerated sample, where silicon nanoclusters merged into micron-sized clusters. Here, methanol solution of white porous silicon was firstly prepared. Then, PBS was added to the methanol solution and methanol was afterwards evaporated at slightly elevated temperature (65°C, 1 mbar, 30 min). After evaporation procedure, total volume of the colloidal solution was less than initial volume of PBS and all methanol was evaporated (we have checked by the absence of the Raman signal from methanol). Then, deionized distilled water was added up to initial volume of PBS in order to maintain constant concentration of native ions in PBS. In this third case, nanoclusters tend to form bigger agglomerates which it is not possible to cut down by further ultrasonic treatment. The results of the DLS experiment in Figure 6a clearly demonstrate that we have successfully obtained three different size distributions of silicon nanoclusters in PBS colloidal solution. Estimated concentration of the filtered samples is about 20 μg/ml, and aged and agglomerated sample maintains original concentration 2 mg/ml. All three types of samples exhibit strong visible photoluminescence which is, compared to standard porous silicon samples, blue shifted to the red-orange spectral region around 600 nm.A note is to be made about ageing of the samples. A freshly prepared and filtered sample of white porous silicon in PBS solution is composed of nanoclusters with sizes well below 100 nm, as depicted in Figure 7. If the sample is left for several weeks at ambient conditions, the nanoclusters get bigger, about 400-nm sized aggregates. In order to avoid this agglomeration, freshly prepared nanoclusters should be used for subsequent biological research. Another possibility is stabilization of nanocrystals via passivating their surface with simple organic compounds; further studies are in progress.

## Conclusions

Colloidal dispersions of porous silicon nanocrystals in methanol, water and PBS show visible luminescence peaked in dependence on the etching conditions at around 700 (standard) and 600 nm (white). In methanol solution, the nanoclusters can be easily separated by size using syringe filters; however, water and PBS dispersions require more sophisticated approach based on filtration, ultrasonication, ageing and combination of two solvents. Prepared PBS dispersions with three different cluster sizes (85, 210 and 1,500 nm) can be subsequently used for biological studies (cytotoxicity and fluorescent label for single-molecule detection in the cell).

## Abbreviations

DLS: Dynamic light scattering; FTIR: Fourier transform infrared (spectra); HF: Hydrofluoric acid; PBS: Phosphate-buffered saline; PL: Photoluminescence; Si-ncs: Silicon nanocrystals; XRD: X-ray diffraction.

## Competing interests

The authors declare that they have no competing interests.

## Authors’ contributions

KH conceived the idea, supervised the research, coordinated the work and wrote the manuscript. EP and MŠ performed etching, ultrasonication and filtration of the silicon nanoclusters and assisted with measurements of DLS and photoluminescence. MŠ measured and interpreted the FTIR spectra, and OC performed measurements of DLS and photoluminescence spectra. JL provided TEM and chemical analysis of the nanoclusters. IP supervised the work and contributed in writing of the manuscript. All authors read and approved the final manuscript.
